# Human Antibody Response to *Anopheles* Saliva for Comparing the Efficacy of Three Malaria Vector Control Methods in Balombo, Angola

**DOI:** 10.1371/journal.pone.0044189

**Published:** 2012-09-24

**Authors:** Laura Brosseau, Papa Makhtar Drame, Patrick Besnard, Jean-Claude Toto, Vincent Foumane, Jacques Le Mire, François Mouchet, Franck Remoue, Richard Allan, Filomeno Fortes, Pierre Carnevale, Sylvie Manguin

**Affiliations:** 1 UMR-MD3, Institut de Recherche pour le Développement, Montpellier, France; 2 UMR-MIVEGEC, Institut de Recherche pour le Développement, Montpellier, France; 3 Medical Service of Sonamet, Lobito, Angola; 4 Laboratoire de Recherche pour le Paludisme, Organisation de Coordination pour la lutte contre les Endémioes en Afrique Centrale, Yaoundé, Cameroun; 5 The Mentor Initiative, Crawley, United Kingdom; 6 National Malaria Control Programme, Luanda, Angola; 7 Institut de Recherche pour le Développement, Montpellier, France; Tulane University School of Public Health and Tropical Medicine, United States of America

## Abstract

Human antibody (Ab) response to *Anopheles* whole saliva, used as biomarker of *Anopheles* exposure, was investigated over a period of two years (2008–2009), in children between 2 to 9 years old, before and after the introduction of three different malaria vector control methods; deltamethrin treated long lasting impregnated nets (LLIN) and insecticide treated plastic sheeting (ITPS) - Zero Fly®) (ITPS-ZF), deltamethrin impregnated Durable (Wall) Lining (ITPS-DL – Zerovector®) alone, and indoor residual spraying (IRS) with lambdacyhalothrin alone. These different vector control methods resulted in considerable decreases in all three entomological (82.4%), parasitological (54.8%) and immunological criteria analyzed. The highest reductions in the number of *Anopheles* collected and number of positive blood smears, respectively 82.1% and 58.3%, were found in Capango and Canjala where LLIN and ITPS-ZF were implemented. The immunological data based on the level of anti-saliva IgG Ab in children of all villages dropped significantly from 2008 to 2009, except in Chissequele. These results indicated that these three vector control methods significantly reduced malaria infections amongst the children studied and IRS significantly reduced the human-*Anopheles* contact. The number of *Anopheles*, positive blood smears, and the levels of anti-saliva IgG Ab were most reduced when LLIN and ITPS-ZF were used in combination, compared to the use of one vector control method alone, either ITPS-DL or IRS. Therefore, as a combination of two vector control methods is significantly more effective than one control method only, this control strategy should be further developed at a more global scale.

## Introduction

Currently vector control for malaria control programs depends largely upon the use of Insecticide Residual Spraying (IRS) of indoor house walls and, or, the more recently developed Long Lasting Insecticide Treated Nets (LLIN). There has been much debate as to the relative advantages and disadvantages of IRS and LLINs [1], [2]. IRS is effective, but normally has to be repeated every 3–6 months, making it difficult to sustain. LLINs have been scaled up across much of Africa through the Roll Back Malaria global partnership since 1998, but obtaining correct and sustained usage of LLINs can be challenging, and there are concerns about LLIN material durability. In some situations, use of other prevention approaches could be suitable. Insecticide Treated Plastic Sheeting (ITPS) has been used in refugees camps to provide combined shelter and vector control [3] and recent WHOPES phase II trial in experimental huts in Burkina Faso showed that permethrin treated plastic sheeting provides the same entomological efficacy as IRS [4]. ITPS has great prospect being long lasting (several years instead of months for IRS) [3], more acceptable as they could be implemented by people themselves instead of an external team, and people could also have some choices in terms of color, material, and therefore ITPS could overcome some of the cultural, social or psychological reject of LLIN. The first trials of ITPS were entomologically successful [4]–[9], but they still had to be tested at a larger scale, such as African villages, for their epidemiological impact on malaria. Durable Lining (Zero Vector®), a newly developed insecticide treated polyethylene shading material, is similar in technology to ITPS, but specifically designed for use on interior walls of traditionally built rural homes, as a replacement for IRS.

The medical service of the Sonamet Company of Lobito, Angola, undertook a vector control program in eight villages of the Balombo area at the request of the national authorities with a comprehensive classical evaluation based on entomological, parasitological and social surveys. In addition, this study addresses a potentially important application of the anti-mosquito saliva antibody responses as immunological biomarker to evaluate and to compare the efficacy of different vector control strategies. As previously described, the saliva of *Anopheles* mosquitoes contains a complex mixture of biologically active proteins which induces the production of specific antibodies such as immunoglobulin G (IgG) that can be used to evaluate individual exposure to *Anopheles* mosquito bites [10]–[17]. Moreover, their usefulness as biomarker tool assessing accurately the efficacy of LLIN has also been reported in population living in a moderate malaria transmission area of Angola [12]. In the present study, IgG responses to whole *Anopheles* saliva were evaluated before and after the introduction of the three vector control strategies: LLIN + ITPS-ZF, IRS alone, and ITPS-DL alone, in children between 2 and 9 years old living in six out of the eight surveyed villages of the malaria-endemic area of Balombo (Angola). The efficacy of each vector control is evaluated and discussed.

## Materials and Methods

### Ethics Statement

This study was conducted in accordance with the Edinburgh revision of the Helsinki Declaration and was approved by the National Malaria Control Program of the Ministry of Health of Angola, the Ethical authority in charge of approving studies on malaria research in Angola. Written consent (signed by the head of each household) was obtained for all individuals enrolled in the study by the SONAMET Company - Malaria Control Program (MCP) which is responsible for malaria surveillance and control amongst company employees and their families.

### Vector control methods

The Balombo program started in 2006 with the objective to develop a malaria control program using and comparing three methods of vector control, including IRS (with lambdacyhalothrin at 25 mg a.i./m^2^), LLIN (PermaNet with deltamethrin at 55 mg a.i./m^2^) used in combination with ITPS-ZF (Zero Fly® treated with deltamethrin at 360 mg a.i./m^2^), and ITPS-DL (ZeroVector® treated with deltamethrin at 170 mg a.i./m^2^). In this study, ITPS-ZF and ITPS-DL were used in a similar manner fixed on the walls of sleeping rooms.

**Figure 1:**
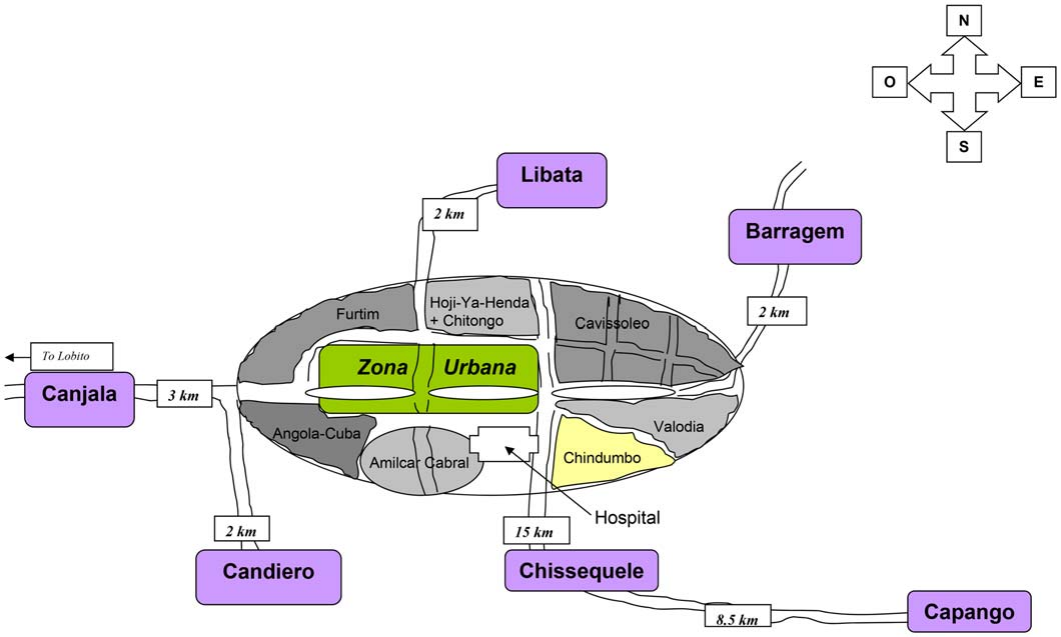
Map of the Balombo area with the localization of the six study sites.

Each of the three vector control methods was implemented in paired-villages in the Balombo area (Table 1). Villages were paired (one high and one medium transmission) to receive one of the three vector control methods (Table 1, Fig. 1) following a parasitological cluster sampling survey in November 2006 based on 557 individuals under 15 years old including 309 females and 248 males randomly chosen. The survey identified Capango, Chissequele, Libata villages as having high malaria transmission (high parasitic index ≥80%), and Canjala, Barragem, Candiero villages as medium transmission (medium parasitic index <80%). The parasitic index was calculated as the percentage of infected people on the total randomly sampled population of each village. In December 2008, Capango and Canjala received the combination (LLIN+ITPS-ZF), Chissequele and Barragem received ITPS-DL alone, and IRS alone was implemented in Libata and Candiero, (with a second IRS round in June 2009). The fourth pair of villages, Caala and Cahata, received LLIN only. However, analysis of samples from these last two villages was excluded from this study as the LLIN alone was not implemented in the same time frame as the three interventions in the other three village pairs, but spaced out in 3 phases during 2008. All houses of the 8 villages received the respective vector control methods.

**Table 1:** Implementation of each vector control method in pair villages (population size) in December 2008.

Villages	Parasitic index^1^	Vector control methods
Capango (∼200 inhabitants)	86%	LLIN + ITPS-ZF
Canjala (∼900 inhabitants)	65%	
Chissequele (∼420 inhabitants)	95%	ITPS-DL (green)
Barragem (∼620 inhabitants)	70%	ITPS-DL (grey)
Libata (∼1,350 inhabitants)	95%	IRS^2^
Candiero (∼660 inhabitants)	63%	

LLIN, Long lasting insecticidal net; ITPS-ZF, insecticide treated plastic sheeting-Zero Fly®; ITPS-DL, Durable wall lining; IRS, Insecticide residual spraying.

1 , Parasitic index (% of infected people) based on a survey done in April 2006.

2 , IRS done twice in Dec 08 and June 09.

### Study area

The six study villages are in the Balombo Municipality, a city of western Angola (12°21′S; 14°46′S), located 600 km southwest of the capital, Luanda (Figure 1). The study area is located at an altitude of 1,200 meters, in the tropical savannah with a rainy season from November to April. The duration of the malaria transmission season varies between 7 and 12 months with a peak between January and May. The most abundant malaria vector in this area was *Anopheles funestus* (Carnevale & Toto, unpublished data).

### Sampling collections

Parasitological cross sectional surveys were conducted every two months from February 2008 to December 2009 on a random sample of households was selected for each survey following a census of each village by the local health worker. Then, a subsample was selected dealing with all children of 2–9 years old in order to obtain the classical “endemic index” and to allow the comparison among villages and periods of the year. For each individual, a thick blood smear was made to assess infection status, *Plasmodium* species, and parasiteamic load. Simultaneously, a drop of blood was taken on a filter paper for further immunological analysis. Two weeks before every parasitological survey, an entomological survey was implemented in each study village. Ten CDC Light Traps were installed in randomly chosen houses (10 houses/village/survey; same houses for each survey) and *Anopheles* densities were calculated as the sum of the mosquitoes collected in the 10 houses/village/survey. In 2008 and 2009, 36 entomological surveys were done (6/village/year) at the same period of the year, corresponding to 60 traps per survey per year, for a total of 360 traps-nights per year. Mosquito species were identified and *Anopheles* specimens analyzed at Yaoundé (Cameroun) for molecular identification of *An*. *gambiae* forms (M or S) [18], and *P. falciparum* infection by circumsporozoite (CSP) ELISA tests [19].

During the first year of the study (2008) and prior to implementation of vector control methods, baseline studies were conducted in each village ( = control year) to obtain main information on vectors, such as *Anopheles* species and infectivity rates of specimens collected by light traps inside houses, as well as prevalence of *Plasmodium* infection and the parasitaemic load in children. This baseline data allowed the comparison before and after the implementation of vector control methods that were implemented from the end of December 2008 onwards.

A total of 11,079 thick blood smears were taken and read at the Medical Department of Sonamet in Lobito during the monitoring period. For the age group of interest, 2–9 years old, the evolution of the classical Endemicity Index group was investigated and for the immunological analysis, 22 blood spots (on Whatman filter paper) per village were made from 22 randomly selected children.

### Extraction of salivary glands

Salivary glands were extracted from uninfected female *An. gambiae* reared in an insectarium (IRD, Montpellier, France) at 27°C and 70%–80% of humidity. Mosquitoes (one week old) were fed with rabbit blood before dissection 2 days later after blood digestion. For dissection, they were anaesthetized at 4°C and placed on ice in a Petri dish. Mosquitoes were then deposited in a drop of PBS 10 mM buffer on a glass slide mounted under the light microscope. The head and the thorax were carefully and progressively separated with needle-tip probe (with gently pressure on thorax) to keep the glands attached to the head. Pairs of salivary glands were collected into 25 µL of PBS 10 mM buffer with protease inhibitor (1/500). After extraction, all salivary glands were pooled and stored at −80°C before immunological testing. Soluble proteins were extracted by cell lysis and the pools were put into liquid nitrogen for 30 seconds and defrosted at room temperature twice. Samples were then centrifuged for 30 min at 20,000 g at 4°C and the supernatant was pooled in a tube.

A large pool of salivary glands (n = 1,578 pairs of glands) was obtained in order to process an equal number of assays including 1,518 samples analyzed and controls. Protein concentrations were measured by Bradford method (Bioscience Bradford Kit).

### Evaluation of human IgG antibody levels by ELISA

Standardized dried blood spots (0.5 cm diameter) were eluted by incubation in 250 µl of phosphate buffered saline (PBS-Tween 0.1%) at 4°C for 48 hours, as previously described [12].

Maxisorp plates (Nunc, Rosklide, Denmark) were coated with the proteins of salivary glands (0.8 µg/mL) in carbonate/bicarbonate buffer for 2 hours and 30 min at 37°C. After washing (with a solution of PBS-Tween 0.1%), the plates were blocked for 45 min at 37°C with 300 µl of Blocking Buffer. Thereafter, each eluate was incubated in duplicate at 4°C overnight at 1/20 dilution (in PBS-Tween 1%). Mouse biotinylated Ab to human IgG (BD Biosciences) was incubated at a 1/1000 dilution at 37°C for 1 hour and 30 min and peroxidase-conjugated streptavidine was added following the same conditions for 1 hour. Colorimetric development was carried out using ABTS (2,2′-azino-bis(3-ethylbenzthiazoline 6-sulfonic acid diammonium) in 50 mM citrate buffer (pH 4) containing 0.003% H_2_O_2_, and absorbance was measured at 405 nm. In parallel, each test sample was assessed in a blank well containing no salivary gland protein (ODn) to measure non specific reactions. A pool of samples was used for the positive control. Negative control sera were obtained from healthy adults living in a non-malaria region in Montpellier (Hérault) and Seillans (Var), France. Individual results were expressed as the ΔOD value: ΔOD = ODx – ODn, where ODx represents the mean of individual OD in both antigen wells, as previously described [12].

### Statistical analysis

Data were analyzed and graphs constructed with GraphPad Prism5® software (San Diego, CA, USA). The non-parametric Mann-Whitney U-test was used for comparison of median Ab levels before and after each vector control intervention, between two vector control measures and villages. The non-parametric Kruskal-Wallis test was used for comparison of medians Ab levels between more than two independent groups and the χ2 test for comparison of two proportions. All differences were considered significant at *P*<0.05.

## Results

### Entomological and parasitological data

During both years, 2008 and 2009, a total of 10 *Anopheles* species were collected in the Balombo area including two main malaria vectors such as *An. funestus* (51.4%), *An. gambiae* (6.4%), along with *An. marshalli* (15.6%), *An. ziemanni* (5.9%), *An. maculipalpis* (15.9%), *An. hancocki* (0.5%), *An. nili* (0.5%), *An. pharoensis* (0.7%), *An. coustani* (1.9%), and *An. tenebrosus* (1.2%). Ten and 8 species were collected in 2008 and 2009 respectively with the absence of *An. nili* and *An. coustani* in 2009.

Two peaks of *Anopheles* densities were found in April 2008 and/or October 2008 for 5 villages, and one village, Libata, showed only one peak at the end of January 2008. Capango had the highest density of *Anopheles* (pre-intervention) with 147 specimens collected from houses in the 2008 baseline survey, compared to just 28 specimens in 2009 (Table 2), showing a reduction of 81.0%. However, the largest reduction of *Anopheles* between 2008 and 2009 occurred in Chissequele, reaching 94.3%, while Barragem experienced the lowest decrease of just 11.1% by 2009 (Table 2). The overall mean reduction of *Anopheles* for all villages combined was 79.1% in 2009, proving the high efficacy of the vector control methods tested. When comparing the data per vector control method, the decrease in vector densities in 2009 varied from 73.2% reduction in villages with ITPS-DL alone (Chissequele - Barragem), to 82.1% in villages using a combination of LLIN and ITPS-ZF (Capango – Canjala), with the intermediate value of 77.7% in villages with IRS (Libata – Candiero). The decrease in *Anopheles* densities was not significantly higher when vector control methods were combined compared to the use of one method only (ITPS-DL or IRS) (Table 2).

**Table 2:** Number and frequency of *Anopheles* specimens collected in each village and for all villages in 2008 and 2009 and density peaks per village.

Villages	Number of *Anopheles* collected^1^		Decrease (%) between 2008 and 2009^2^		*Anopheles* density peaks, months, year, total collected (%)
	2008	2009			
Capango (n = 175)	147 (84%)	28 (16%)	81.0	82.1	April 08, n = 76 (43%)
					Oct 08, n = 53 (30%)
Canjala (n = 42)	37 (88%)	5 (12%)	86.4		April 08, n = 18 (43%)
Chissequele (n = 56)	53 (95%)	3 (5%)	94.3	73.2	April 08, n = 19 (34%)
					Oct 08, n = 11 (20%)
					Nov 08, n = 13 (23%)
Barragem (n = 34)	18 (53%)	16 (47%)	11.1		Oct 08, n = 5 (15%)
					Sept 09, n = 7 (21%)
Libata (n = 57)	46 (81%)	11 (19%)	76.1	77.7	Feb 08, n = 24 (42%)
Candiero (n = 58)	48 (83%)	10 (17%)	79.2		Feb 08, n = 14 (24%)
					April 08, n = 23 (40%)
**TOTAL (n = 422)**	**349 (83%)**	**73 (17%)**		**79.1**	

1 , Number of *Anopheles* collected by CDC light traps in 10 houses per village once every 2 months from Feb. 2008 to Dec. 2009.

2 , χ^2^ values: between 82.1% and 73.2%, χ^2^ = 1.576 (p = 0.209, NS); between 82.1% and 77.7%, χ^2^ = 0.515 (p = 0.473, NS); between 77.7% and 73.2%, χ^2^ = 0.261 (p = 0.609, NS), (NS: non-significant).

Peaks of *P. falciparum* infection rates in the human population were mainly found in February or April for both years in all villages, except Candiero which showed its 2008 peak in October (Table 3). Significant decreases occurred in the number of *Plasmodium* positive blood smears between 2008 and 2009 with a reduction ranging from 42.4% in Chissequele to 61.7% in Capango. Of 2,706 and 2,218 blood samples of 2–9 years old children collected in 2008 and 2009 respectively, for all villages combined, the overall number of positive blood smears decreased by 54.8% after implementing vector control methods (Table 3). When comparing the decrease of infected blood smears in villages with different vector control method, results ranged from 51.0% in ITPS-DL villages (Chissequele - Barragem) to 53.7% and 58.3% in villages with respectively IRS (Libata – Candiero) and LLIN and ITPS-ZF (Capango – Canjala). Although the decrease was higher in the latter case, differences between vector control methods were not significant (Table 3).

**Table 3:** Number of positive blood smears on the total of collected samples (frequency of positive blood smears) for 2–9 years old children in each village in 2008 and 2009, and decrease (%) in number of positive blood smears between 2009 and 2008.

Villages	Number of positive blood smears on total of collected samples (%)		Decrease (%) between 2008 and 2009^1^		Peaks of infection rate (% positive blood smears)	
	2008	2009			2008	2009
Capango	84/258 (32.6%)	38/303 (12.5%)	61.7	58.3	April (51.1%)	April (22.2%)
Canjala	255/539 (47.3%)	79/356 (22.2%)	53.1		February (53.5%)	February (40.4%)
Chissequele	124/421 (29.5%)	68/400 (17.0%)	42.4	51.0	February (38.6%)	February (28.4%)
Barragem	142/382 (37.2%)	51/333 (15.3%)	58.9		February (57.1%)	April (27.1%)
Libata	307/618 (49.7%)	85/403 (21.1%)	57.5	53.7	February (63.6%)	February (32.7%)
Candiero	141/488 (28.9%)	70/423 (16.5%)	42.9		October (34.2%)	February (32.8%)
TOTAL	1053/2706 (38.9%)	391/2218 (17.6%)		54.8		

1 , χ^2^ values: between 58.3% and 51.0%, χ^2^ = 0.33 (p = 0.56, NS); between 58.3% and 53.7%, χ^2^ = 0.17 (p = 0.68, NS); between 53.7% and 51.0%, χ^2^ = 0.03 (p = 0.85, NS), (NS: non-significant).

### Evaluation of anti-saliva IgG response before and after vector control implementation

**Figure 2:**
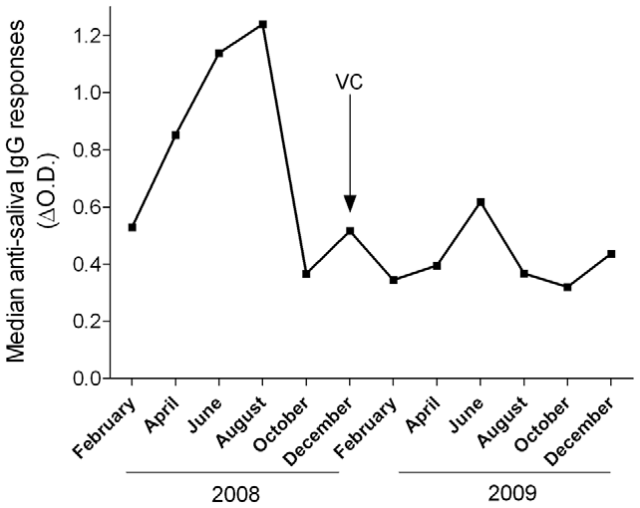
Evolution of the median values of the IgG antibody response to *Anopheles* saliva for all 6 villages combined according to the survey period in 2008 and 2009 (VC: vector control methods implemented in December 2008).

**Figure 3:**
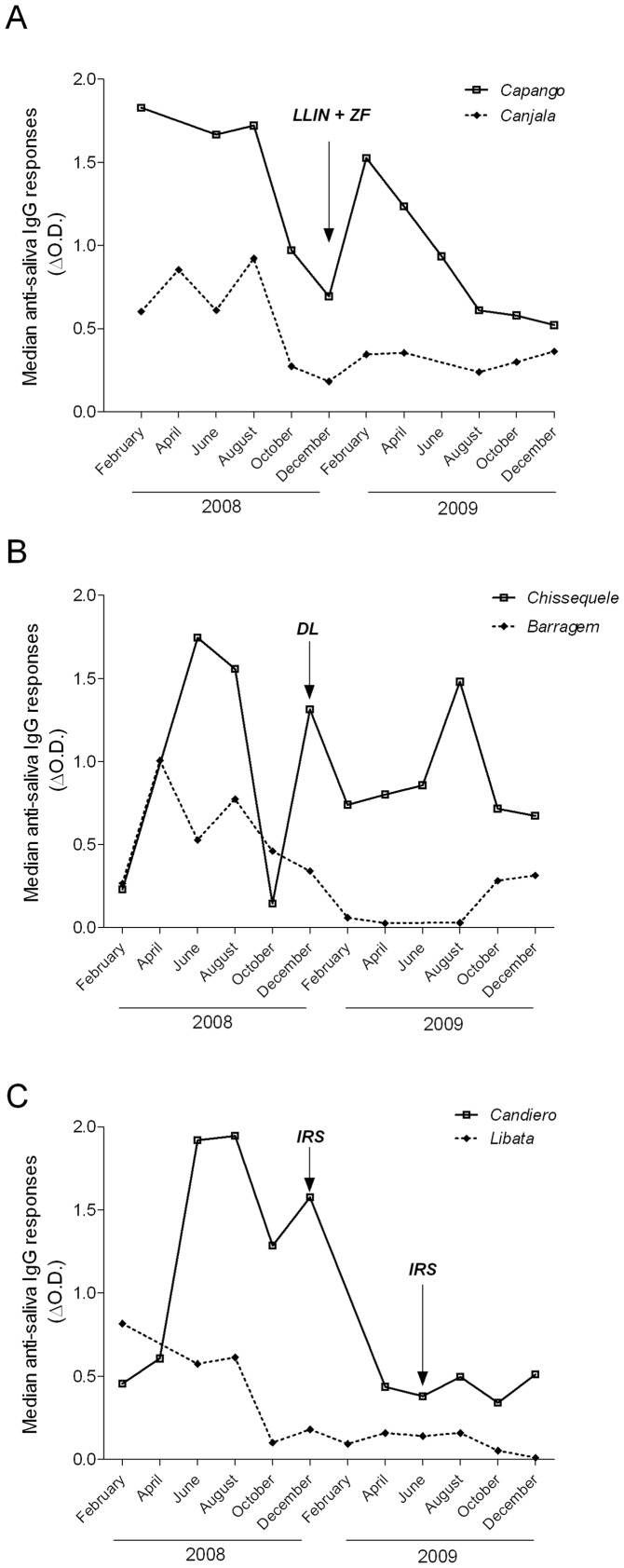
Evolution of the median values of the IgG antibody response to *Anopheles* saliva for each vector control method according to the survey period in 2008 and 2009. A, Insecticide treated plastic sheeting-durable wall lining (ITPS-DL); B, Long lasting insecticide net (LLIN) and Zero fly (ITPS-ZF); C, Insecticide residual spraying (IRS) done in December 2008 and June 2009 (Not available data for 6 surveys: April 2008 for Capango, Chissequele and Libata; February 2009 for Candiero; June 2009 for Canjala and Barragem).

In 2008, before the introduction of vector control methods, the intensity of exposure to mosquito bites was high and varied by season with a peak of IgG antibody (Ab) response to *Anopheles* saliva in August followed by a significant decrease in October (end of dry season) and a small peak in December (Fig. 2). The major peak of anti-saliva IgG Ab in August (Fig. 2) corresponds to the late part of the peak of infection rate in April (Table 3). However, when considering the IgG Ab response to *Anopheles* saliva for each village (Fig. 3), there is no specific trend as peaks of *Plasmodium* infection rate are not clearly related to a specific level of Ab anti-*Anopheles* saliva. After implementation of the vector control methods in late December 2008, the overall median values of the IgG Ab were significantly reduced (p<0.0001), although a small peak of IgG occurred in June 2009 (end of rainy season) (Fig. 2). The median values of specific IgG levels were significantly lower in 2009 than in 2008 for all campaigns and in all villages (p<0.001), except for Chissequele (NS, p = 0.73), showing a significant drop in the intensity of mosquito bites due to the efficacy of vector control methods (Table 4).

**Table 4:** Significance of the implementation of each vector control method on IgG antibody response to *Anopheles* saliva in each village by comparison of years 2008 and 2009 and specific surveys in 2008 and 2009.

Vector control methods	LLIN+ITPS-ZF		ITPS-DL		IRS	
Villages	Capango	Canjala	Chissequele	Barragem	Libata	Candiero
Surveys	P value					
2008/2009	**	**	NS	**	**	**
Dec 08/Feb 09	**	*	*	**	NS	**
Feb 08/Feb 09	NS	*	NA	NS	**	NS
Dec 08/Dec 09	NS	**	**	NS	**	**

LLIN+ITPS-ZF, ITPS-DL, and IRS.

NS, non significant;

* , significant at p<0.05;

** , significant at p<0.001;

NA, non applicable.

The evolution of the median values of the specific IgG Ab for each vector control method is presented in Figure 3. The comparison of the evolution of the IgG response in children of Canjala and Capango, with LLIN and ITPS-ZF, showed two peaks in February–April and August 2008. A decrease was then observed in October–December 2008, followed by a peak in February 2009, then by a very significant or a slight decrease in Capango and Canjala respectively (Fig. 3 A). The overall decrease of the specific IgG level presented in each village between 2008 and 2009 was significant (p<0.001, Table 4).

In Barragem and Chissequele, where ITPS-DL was implemented, the level of anti-saliva IgG Ab among the children of Barragem showed a similar pattern in 2008 to the other villages, with peak values between April and August 2008, followed by a decrease in October 2008 (Fig. 3B). However, the pattern differed according to the village. In Barragem, the level of IgG Ab steadily decreased to be almost zero from April–August 2009. This decrease observed after the introduction of ITPS-DL in December 2008 was significant (p<0.001, Table 4). The slight increase in the IgG Ab found in October and December 2009 was not significant (Table 4). In Chissequele village, the evolution of IgG level showed 2 peaks in 2008, one in June–August and a second one in December. After implementation of ITPS-DL, the IgG values were significantly reduced (p<0.05, Table 4), but a peak of IgG in August 2009 reached a high level and the overall evolution of Ab for both years was non-significant (Table 4).

In Candiero and Libata, IRS was implemented twice (December 2008 and June 2009). In 2008, the evolution of the anti-saliva IgG response presented two peaks in Candiero, June–August and December (Fig. 3C). This pattern is followed by a significant decrease from February (p<0.001, Table 4) onwards, with very small fluctuations occurring through 2009 (Fig. 3C). In Libata, the highest level of specific IgG occurred from February to August 2008, then, the median IgG values reduced to a low level after implementation of IRS. The overall values of anti-saliva IgG response in 2009 were significantly lower than in 2008 for each village (p<0.001, Table 4).

**Figure 4:**
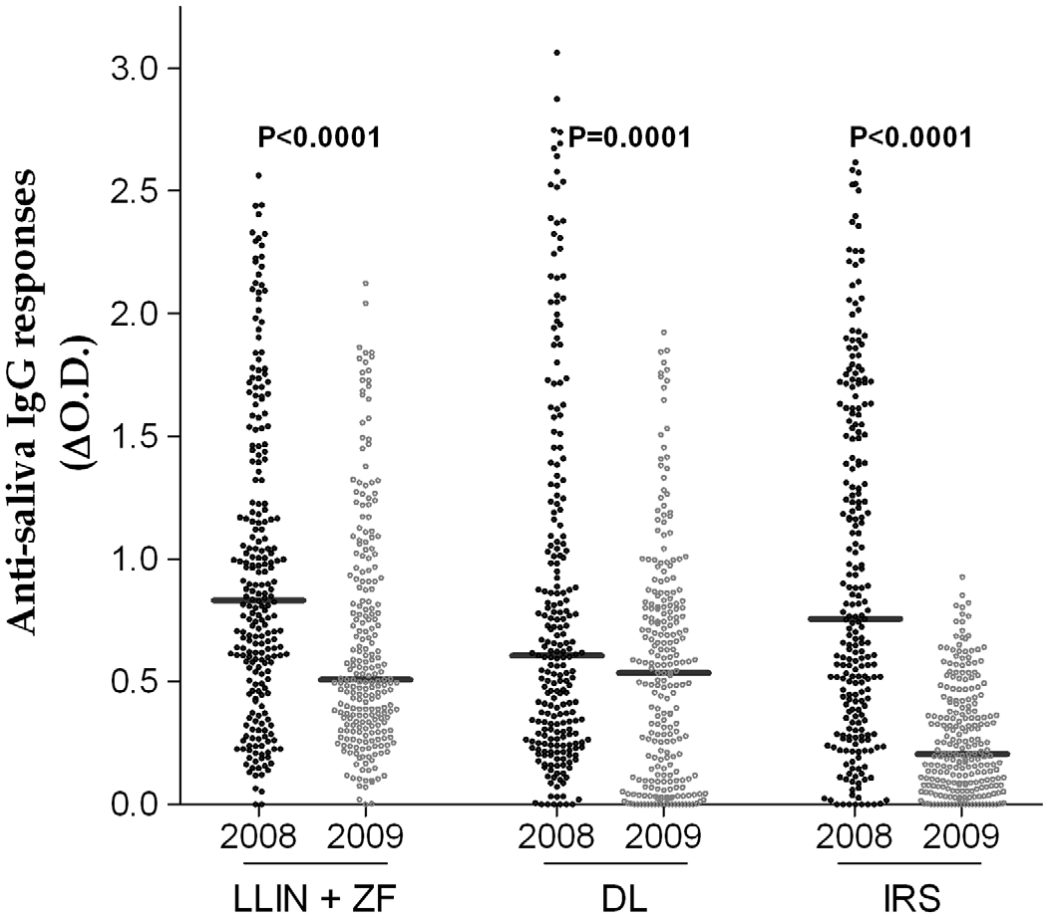
Comparison of median values of the IgG antibody response to *Anopheles* saliva obtained before and after implementation of each vector control method respectively in 2008 and 2009. LLIN+ITPS-ZF (Canjala, Capango); ITPS-DL (Barragem, Chissequele) ; IRS (Libata, Candiero).

The comparison of anti-saliva IgG levels for each vector control methods showed considerable reduction in 2009 compared to 2008 (Figure 4). Indeed, specific IgG levels were significantly lower in 2009 in LLIN+ITPS-ZF and IRS villages (p<0.0001), as well as in villages with ITPS-DL (p = 0.0001). However, the largest decrease in specific IgG Ab response among children was observed in Libata/Candiero where IRS was implemented (Fig. 4).

## Discussion

The present study investigated the human IgG Ab response to *Anopheles* whole saliva over two years (2008–2009), in children between 2 to 9 years old, before and after the introduction of three different vector control methods, LLIN + ITPS-ZF, ITPS-DL alone, and IRS alone, in three-paired villages of Balombo (Angola).

Before the introduction of vector control methods, anti-saliva IgG responses considerably varied according to the season. These variations were village-dependent and clearer in high malaria transmission villages (Capango, Chissequele and Libata). Globally, specific anti-whole *Anopheles* saliva IgG response peaked in April–June and in December (minor peak), preceded by entomological and parasitological peaks in February–April (major) and October–December (minor). These results showed an association between entomological/parasitological data and anti-*Anopheles* saliva IgG Ab levels in Balombo and confirmed previous data obtained in a low transmission area of Angola where *An. gambiae* was the principal vector of malaria pathogens [12]. However, individuals of the present study area were mainly exposed to the bites of *An. funestus* which was the principal malaria vector accounting for 51.4% of all *Anopheles* specimens collected during the study period compared to *An. gambiae* (6.4%) (Carnevale & Toto, unpublished data). Whole *An. gambiae* salivary gland antigens were then successfully used to assess specific IgG levels in children exposed to *An. funestus* bites. This was possible due to biologically active salivary proteins shared by these two related major malaria vectors in south-Saharan Africa [20]. Indeed, saliva of mosquitoes, as well as of other related arthropods, contains a range of molecules that are biochemically and functionally different. Some of these components are specific, while others may be common to several species, genera, families of mosquitoes [21]. This fact points to the relevance of developing a unique salivary biomarker of bites common to the major anopheline vector species. Such a tool could potentially be as easily used in field conditions, as current rapid diagnostic tests are, in order to evaluate the exposure of human populations to major African malaria vector bites, as previously suggested [22].

After the introduction of vector control methods (in December 2008), the overall results showed considerable decreases, within one year, in all three entomological, parasitological and immunological criteria analyzed, regardless of the vector control method used. These results point to an association between the entomo-parasitological data and anti-saliva IgG Ab levels and a high effectiveness of the three vector control methods in the studied population of children. They confirm the validity of this anti-vector Ab parameter as an immuno-epidemiological indicator for malaria vector control effectiveness as previously described in a urban area of Angola [12]. Independent of the level of endemicity, indoor spraying (IRS) conferred an efficient protection against mosquito bites as shown in Figure 4. However, when considering all three criteria, as the number of *Anopheles* and positive blood smears, and the levels of specific IgG, the combined use of LLIN and ITPS-ZF proved to be even more effective than the use of one vector control method alone, either ITPS-DL or IRS. Therefore, as two vector control methods used in combination have proven to be significantly more effective than one method alone, this strategy of combining control methods should be further and more widely developed in areas of similar perennial and high malaria transmission. Combining anti-vector tools can reduce transmission and hence malaria burden more rapidly than may be feasible with one method alone [23]. In addition, it represents a way to delay the emergence of insecticide resistance by using different classes of insecticide for LLIN and IRS or ITPS-DL [24]. Analyzed at the village level, immunological data based on the level of anti-saliva IgG Ab in children decreased significantly from 2008 to 2009 in all villages, except in Chissequele. The non-effectiveness of ITPS-DL in reducing the human-*Anopheles* contact level was confirmed by the lower decrease in positive blood smears (42.4%) while a considerable decrease was concomitantly observed in the density of *Anopheles* specimens collected (94.3%). Discrepancies between anti-*Anopheles* saliva IgG levels and entomological data have been reported in low-exposure/transmission areas of Angola [11], [12] and Senegal [25] and were linked to a difference of sensitivity between the two methods. In Chissequele, a high malaria transmission area, the most probable hypothesis explaining this observation is that the ITPS-DL was not properly maintained or used by households, and therefore the coverage was not sufficient to achieve adequate reduction in *Anopheles* mosquitoes and human contact, and infection. This hypothesis is supported by results of a recent survey in Chissequele (26/08/2011) that showed that of a total of 119 housing provided with ITPS-DL in 2008, 78% had discarded it (Carnevale et al., unpublished data). Overall, malaria prevention results achieved with ITPS-DL and IRS were similar. However, ITPS-DL did not require repeat applications as did IRS, during the study period, but IRS cannot be removed as ITPS-DL (or LLIN) as shown in Chissequele.

The overall results showed a better impact of the simultaneous implementation of deltamethrin treated LLIN and ITPS-ZF on reducing the density of *Anopheles*, the human-*Anopheles* contact level and the prevalence of *Plasmodium* than the use of either ITPS-DL or IRS alone. However, IRS with lambdacyhalothrin showed a high efficacy in reducing the human-*Anopheles* contact. They also suggest that antibodies (IgG) specific to *An. gambiae* whole saliva could constitute an efficient and reliable indicator for evaluating and comparing the effectiveness of different malaria vector control methods or strategies. Such an indicator could also represent an alternative to classical entomological-parasitological monitoring methods for measuring and following the effectiveness of vector control strategies used by the National Malaria Control Programmes in Africa.
